# Factors associated with a change in smoking habit during the first COVID-19 lockdown: an Italian cross-sectional study among ever-smokers

**DOI:** 10.1186/s12889-022-13404-5

**Published:** 2022-05-25

**Authors:** Elena Munarini, Chiara Stival, Roberto Boffi, Fabio Lugoboni, Chiara Veronese, Biagio Tinghino, Gianna Maria Agnelli, Alessandra Lugo, Silvano Gallus, Rosaria Giordano

**Affiliations:** 1 Pulmonology Unit, Fondazione IRCCS Istituto Nazionale dei Tumori, 20133 Milan, Italy; 2 Department of Environmental Health Sciences, Istituto di Ricerche Farmacologiche Mario Negri IRCCS, 20156 Milan, Italy; 3 Department of Medicine, Addiction Medicine Unit, Verona University Hospital, 37134 Verona, Italy; 4 Alcohol and New Addictions Unit, ASST Brianza, 20871 Vimercate, Italy; 5 Occupational Health Unit, Clinica del Lavoro “L. Devoto”, Fondazione IRCCS Ca’ Granda Ospedale Maggiore Policlinico, 20122 Milan, Italy; 6 Department of Neuroscience, Biomedicine and Movement, University of Verona, 37129 Verona, Italy

**Keywords:** COVID-19, Pandemics, Smoking cessation, Smoke, Tobacco use disorder, Psychological stress

## Abstract

**Background:**

The COVID-19 pandemic and the lockdown period lasted from March to May 2020, resulted in a highly stressful situation yielding different negative health consequences, including the worsening of smoking habit.

**Methods:**

A web-based cross-sectional study on a convenient sample of 1013 Italian ever smokers aged 18 years or more was conducted. Data were derived from surveys compiled by three different groups of people: subjects belonging to Smoking Cessation Services, Healthcare Providers and Nursing Sciences’ students. All institutions were from Northern Italy. The primary outcome self-reported worsening (relapse or increase) or improvement (quit or reduce) of smoking habit during lockdown period. Multiple unconditional (for worsening) and multinomial (for improving) logistic regressions were carried out.

**Results:**

Among 962 participants, 56.0% were ex-smokers. Overall, 13.2% of ex-smokers before lockdown reported relapsing and 32.7% of current smokers increasing cigarette intake. Among current smokers before lockdown, 10.1% quit smoking and 13.5% decreased cigarette intake. Out of 7 selected stressors related to COVID-19, four were significantly related to relapse (OR for the highest vs. the lowest tertile ranging between 2.24 and 3.62): fear of being infected and getting sick; fear of dying due to the virus; anxiety in listening to news of the epidemic; sense of powerlessness in protecting oneself from contagion. In addition to these stressors, even the other 3 stressors were related with increasing cigarette intensity (OR ranging between 1.90 and 4.18): sense of powerlessness in protecting loved ones from contagion; fear of losing loved ones due to virus; fear of infecting other.

**Conclusion:**

The lockdown during the COVID-19 pandemic was associated with both self-reported relapse or increase smoking habit and also quitting or reduction of it.

**Supplementary Information:**

The online version contains supplementary material available at 10.1186/s12889-022-13404-5.

## Background

The coronavirus disease (COVID-19 pandemic) is an ongoing global pandemic associated by severe acute respiratory syndrome coronavirus 2 (SARS- CoV-2). The outbreak was first identified in December 2019 in Wuhan, China [1]. The World Health Organization (WHO) declared the outbreak a Public Health Emergency of International Concern on 30 January 2020 and a pandemic on 11 March 2020 [2]. To contain and combat the spread of the virus, starting on 11 March 2020, the Italian Government adopted restrictive measures throughout the country: the obligation to wear a surgical mask, the prohibition on leaving home and travel except for urgent reasons and ordered the closure of all facilities in which people gather, such as restaurants, cinemas and museums. Furthermore, smart working and the remote school were activated. This period, called “lockdown”, lasted in its strictest mode until May 18, 2020 [3]. Once the lockdown was over, many measures to contain the pandemic were maintained in the following months; neither during lockdown or later there was restriction of tobacco purchase. In this paper we refer strictly to the lockdown period that characterized the first phase of the pandemic in Italy, as it was the one with the strictest restrictions.

Recent studies have shown that the lockdown confinement and the simultaneous closure of all leisure and socialization activities has important health, economic, environmental and social consequences [4].

Tobacco consumption is the single greatest preventable cause of death in the world today [5] and an important risk factor for disease severity and worse outcomes in COVID-19 [6–9]. The worsening of life condition due to the changes produced by lockdown suggests that smokers can be likely to increase smoking and that ex-smokers may relapse [10], with further, important health consequences..

Many changes occurred in lifestyle and daily activities such as work routine, sleeping habits, physical inactivity and nutritional habits [11, 12]. These aspects, in turn, may have induced in people a change in their smoking behavior [13].

Regarding psychiatric pathologies, the current evidence suggests that a psychiatric epidemic is occurring parallel to the COVID-19 pandemic, which is increasingly worrying to the global healthcare community [14]. An increase in several psychiatric disorders including anxiety, sleep disorders and panic has been detected to a larger extent in people who already had a previous vulnerability to psychiatric pathologies [15, 16]. Since nicotine produces acute anxiolytic effects [17] smokers who experience elevated anxiety symptoms are more likely to use smoking as a means of regulating their mood [18].

Only a few studies have thoroughly investigated the change in smokers’ and ex-smokers’ behaviors associated to lockdown and the influencing variables. The results reported to date are rather ambivalent: Caponnetto et al. [19] found a slight decrease in cigarette consumption and a slight increase in e-cigarette use, whereas Bommele et al. examined the role of stress in influencing the amount of cigarettes smoked and found that moderate and high levels of stress can induce both a reduction and an increase in smoking [20]. Stress, however, is certainly an important factor to consider, given its ability to induce an increase in smoking [21].

Starting from this background we wanted to investigate if ever smokers reported getting worse in smoking habit (i.e., relapse for ex-smokers before lockdown or increase in the number of cigarettes per day for current smokers before lockdown), or having improved it (i.e., smoking cessation or reduction in the number of cigarettes per day for current smokers before lockdown). Moreover, we aimed to exploratorily evaluate the relationship between selected specific stressors and changes in smoking behavior due to COVID-19. We conducted this analysis on three different Northern Italy populations, assuming that they could have been particularly hit by the confinement measures: heavy ever smokers attending Smoking Centers Services (SCSs), Healthcare Providers (HPs), and Nursing Sciences’ students.

## Material and methods

We conducted a web-based cross-sectional study on a sample of Italian ever smokers aged 18 years or more to observe the impact of COVID-19 lockdown on smoking habits. The web-based survey was launched by e-mail on May 15th 2020, and remained available for 1 month.

### Study population and sampling procedure

Data were derived from a sample of 1013 Italian adults aged 18-80 years from northern Italy. Study participants were a convenience sample of ever smokers recruited among three different groups of the population: i) subjects referred to Smoking Cessation Services (SCSs), ii) Healthcare Providers (HPs), and iii) Nursing Sciences’ students. The SCSs included were the National Cancer Institute (Milan, Italy), the Alcohol and New Addictions Unit of the Major Hospital of Milan, (ASST Brianza, Vimercate, Italy) and the Unit of Addiction Medicine (Integrated University Hospital of Verona, Verona, Italy). The HPs involved belonged to the National Cancer Institute (Milan, Italy) and to the Integrated University Hospital of Verona. Nursing Sciences’ students were recruited from the University of Verona.

To recruit the sample, participants have been contacted by email: 943 email of the subjects referred to Smoking Cessation Services (SCSs), 817 email of Healthcare Providers (HPs), and 293 email of Nursing Sciences’ students. The availability of the email addresses of these last two groups was there because of their participation in several past years in educational events on smoking cessation organized by the anti-smoking services of Milan and Verona. Therefore subjects from HPs and Students were both ever smokers and never-smokers. Only ever smokers were asked to answer.

An online survey, composed of 26 multiple-choice questions and eight open questions, for a total of 34 questions, was created on Survey Monkey. The link to the online questionnaire was sent by e-mail to all current and ex-smokers who had attended an SCS before the lockdown, and to HPs and students of the hospitals involved in the study.

The survey targeted ever-smokers (respondents who reported smoking ≥100 cigarettes in their lifetime) [22]; the link to the questionnaire was sent to 2053 subjects via mail. They were then asked whether they were current smokers (respondents who reported smoking ≥100 cigarettes in their lifetime and has smoked in the last 28 days) or ex-smokers (respondents who reported smoking greater than 100 cigarettes in their lifetime but has not smoked in the last 28 days) on the date of completion of questionnaire. The time needed to complete it was approximately 15 min, and each subject was able to fill in the online survey only once. Participation in the survey was voluntary, anonymous and without remuneration. Participants expressed their consent to participate in the study by completing the corresponding field in the online questionnaire.

### Independent variables

The survey included questions regarding socio-demographic characteristics (sex, age, marital status, children’s presence, educational qualification and employment status). Instead of educational qualification, a specific question about current profession was asked to the HPs.

The survey comprised a section that included a combination of 21 stressors potentially associated by the COVID-19 pandemic and the first lockdown in Italy. We divided the 21 stressors into four groups considering their common characteristics (Table 1). To select the stressors, we referred to the work of Taylor [23], whose research and clinical observations suggested that during times of pandemics many people experience fear and anxiety referring to the following areas of discomfort: fear of becoming infected, fear of coming into contact with potentially contaminated objects or surfaces, fear of strangers who may be carrying infections (e.g. related xenophobia), fear of the socioeconomic consequences of the pandemic (e.g. job loss), compulsive control, and seeking reassurance about possible pandemic-related threats and symptoms of traumatic stress about the pandemic (e.g., nightmares and intrusive thoughts). We qualitatively select the areas of most interest, excluding those that were studied in other parts of the questionnaire.

Table 1 The impact of lockdown on mood was evaluated through a question based on a seven point Likert scale (ranging from 1 “my mood is much improved” to 7, “my mood is much worse”) that explored the difference between the period before and during lockdown. Sleep quality was evaluated by a four choice question: very good, good enough, bad enough, and very bad.

The psychological aspects and the emotional impact related to lockdown were investigated using two Italian validated scales: the General Health Questionnaire (GHQ-12) was used to investigate respondents’ quality of life [24–27] and the State-Trait Anxiety Inventory Y-1 (S.T.A.I. Y-1) to investigate state anxiety during lockdown [28, 29].

**Table 1:** List of the 21 stressors divided in four groups

***Stressors related to COVID-19***	***Stressors due to isolation***	***Social issues resulting from lockdown***	***Professional and school issues***
Fear of being infected and getting sick;	Impossibility/fear of approaching health facilities while needed;	Fear of being judged by the neighbors for exits dictated by reasons of need, work, etc.;	Fear of losing livelihoods/being fired;
Fear of infecting others;	Fear of separating from loved ones for the quarantine regime;	Fear of being socially excluded due to the origin of regions affected by the virus;	Reduction of work and economic opportunities due to the need to stay at home with children for the closure of schools;
Sense of powerlessness in protecting oneself from contagion;	Sense of powerlessness due to isolation;	Worsening of family relationships during quarantine;	Reduction of economic opportunities for redundancy fund (for company limitations from COVID-19);
Sense of powerlessness in protecting loved ones from contagion;	Boredom due to isolation;	Fear that nothing will be like before lockdown (9th March 2020).	Difficulty in managing children and teaching online;
Fear of losing loved ones due to virus;	Feelings of loneliness due to isolation.		Difficulty in adapting to smart working.
Fear of dying due to the virus;			
Anxiety in listening to news of the epidemic.			

### Main outcome variables

The survey had a section dedicated to smoking behavior, divided in two subsections: one for ex-smokers and one for current smokers. Ex-smokers were asked to report the date they quit to further divide them into ex-smokers for more than 12 months and ex-smokers for less than 12 months. The section for ex-smokers included questions about the age at starting smoking, the number of cigarettes smoked per day and cravings related to the lockdown. The section for current smokers included questions about age when starting smoking, the number of cigarettes smoked per day before lockdown, the number of cigarettes smoked per day during lockdown and the idea of quitting smoking related to the beginning of lockdown. Moreover, current smokers were asked whether they were ex-smokers before lockdown and relapsed during the lockdown and, in case of relapse, about their smoking cessation’ year and month.

Through self-reporting, we defined worsening smoking habit during lockdown if i) individuals relapsing in smoking vs. those not relapsing among ex-smokers before lockdown, ii) individuals increasing vs. those not increasing the number of cigarettes per day smoked among current smokers before lockdown. We defined improving smoking habit during lockdown if: i) individuals reported have quitted smoking and ii) individuals reported to have kept smoking during lockdown, but decreased the total number of cigarettes per day smoked, both compared with those who did not improve (not quit and not decrease the number of cigarettes per day) their smoking habit among smokers before lockdown.

### Statistical analysis

A power calculation was not a priori performed. By protocol we hypothesized to obtain the filled in questionnaire for 400 of the 800 invited subjects from the SCS. No hypothesis was assumed for the other centers. A post-hoc power calculation was performed with 962 individuals, assuming alpha = 0.05 and the prevalence of people worsening smoking habit of 30%, our data have sufficient statistical power (> 0.80) to obtain a statistically significant odds ratio (OR) of 1.50 in a dichotomous variable with equally distributed categories.

We considered descriptive statistics including relative frequency (%) for categorical variables and mean and standard deviation (SD) for continuous variables. Multiple logistic regression models after adjustment for sex, age group and group of participants (SCSs, HPs and students) were used to derive odds ratios (ORs) and corresponding 95% confidence intervals (CIs) for worsening smoking habits during lockdown. Multinomial logistic regression models after adjustment for the same covariates were used to derive ORs for improving smoking habits during lockdown. The Statistical significance level (alpha) was set to 0.05. After checking the linearity assumption for age using Box-Tidwell test for all the models, we considered age in categories.

All statistical analyses were performed using the software SAS, version 9.4 (Cary, North Carolina, USA).

## Results

### Survey respondents

**Fig. 1:**
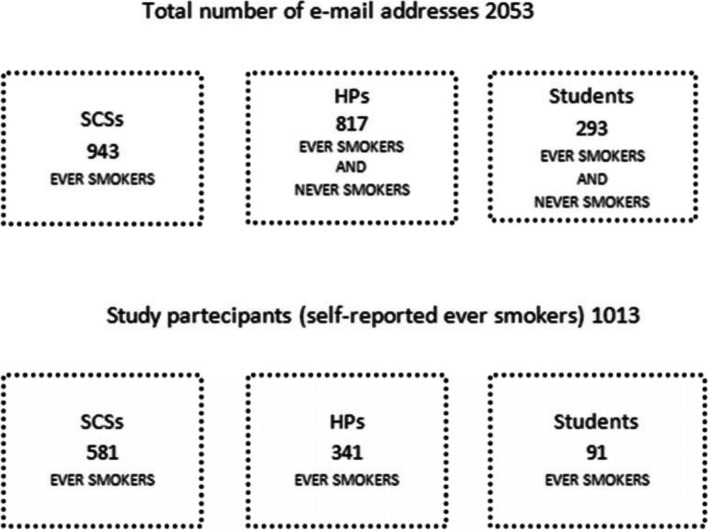
Total number of e-mail addresses and respondents. SCSs: Smoking Centers Services. HP: Healthcare Providers

Of 2053 subjects that received the link to the questionnaire, 1013 responded (Fig. 1). Fifty-one were excluded from data analysis because they did not report their smoking status before lockdown. Thus, a total of 962 participants were included in this study. Response rate was respectively 62% for SCSs, 42% for HPs and 31% for Students.

### Sociodemographic variables and smoking habits before and during the COVID-19 lockdown

Sociodemographic characteristics and smoking habits before and during the COVID-19 lockdown are presented in Table 2. The total sample of 962 ever smokers (56.0% ex-smokers and 44.0% smokers before lockdown) had a mean age of 48.6 years (SD: 13.8); 58.9% were women. The majority of respondents belonged to the SCS group (59.5%).

**Table 2:** Baseline characteristics of the 962 ever smokers before COVID-19 lockdown, overall and by their smoking habit

Characteristics	Total N (%)	Ex-smokers N (%)	Current smokers N (%)
Total	962 (100)	539 (100)	423 (100)
Sex			
Men	395 (41.1)	235 (43.6)	160 (37.8)
Women	567 (58.9)	304 (56.4)	263 (62.2)
Age			
< 39	240 (24.9)	113 (21.0)	127 (30.0)
40-54	346 (36.0)	197 (36.6)	149 (35.2)
55+	376 (39.1)	229 (42.4)	147 (34.8)
Mean (SD)	48.6 (13.8)	50.0 (13.2)	46.7 (14.3)
Marital status			
Married/cohabitants	531 (55.2)	331 (61.4)	200 (47.2)
Divorced/separated	134 (13.9)	68 (12.6)	66 (15.6)
Widowed	30 (3.1)	12 (2.2)	18 (4.3)
Single	267 (27.8)	128 (23.8)	139 (32.9)
Group of participants			
SCS	572 (59.5)	354 (65.7)	218 (51.5)
Healthcare provider	310 (32.2)	157 (29.1)	153 (36.2)
Students	80 (8.3)	28 (5.2)	52 (12.3)

*SCS* Smoking Cessation Service, *SD* Standard deviation

### Relapse in ex-smokers and worsening of current smokers before lockdown

Table 3 shows the distribution of ex-smokers and current smokers before lockdown according to a self-reported worsening of their smoking habit. We found that 13.2% of the 539 ex-smokers before lockdown stated to have relapsed during lockdown and that 32.7% of the 416 current smokers reported to have increased the number of cigarettes per day during the lockdown (mean increase, 6.2 cigarettes per day; SD, 4.5). Relapse was less frequently reported with increasing pack-years (*p* for trend = 0.033). Relapses were more frequently reported among widowed (compared married/cohabitants; OR = 4.20; 95% CI, 1.15-15.3) and ex-smokers who quit after 2018 (OR = 7.22; 95% CI: 3.53-14.8). No relationship between sex, age and group of participants, and relapse was observed.

**Table 3:** Distribution of the 539 ex-smokers and the 416^a^ current smokers before lockdown (multiple unconditional logistic regression analysis)

Characteristics	Total number of ex-smokers before lockdown^**c**^	People relapsing during lockdown		Total number of current smokers before lockdown^**c**^	People increasing n° of cigarettes per day during lockdown	
		%	OR^b^ (95% CI) Relapse vs not relapse		%	OR^b^ (95% CI) Increase vs not increase
Total	539	13.2		416^a^	32.7	
Sex						
Men	235	12.3	1.00^d^	160	27.5	1.00^d^
Women	304	13.8	1.32 (0.77–2.24)	256	35.9	**1.56 (1.00–2.44)**
Age group (years)						
18–39	113	11.5	1.00^d^	124	31.5	1.00^d^
40–54	197	15.7	1.21 (0.57–2.55)	147	28.6	0.73 (0.40–1.34)
55+	229	11.8	0.84 (0.39–1.79)	145	37.9	1.11 (0.61–2.03)
*p* for trend			0.423			0.481
Group of participants						
SCSs	354	14.4	1.00^d^	215	33.5	1.00^d^
Healthcare provider	157	11.5	0.70 (0.38–1.27)	149	34.2	0.96 (0.60–1.53)
Students	28	7.1	0.40 (0.08–1.99)	52	25.0	0.55 (0.24–1.28)
Marital status						
Married/cohabitants	331	11.8	1.00^d^	196	35.7	1.00^d^
Divorced/separated	68	16.2	1.43 (0.68–3.01)	66	37.9	0.95 (0.52–1.72)
Widowed	12	33.3	**4.20 (1.15–15.3)**	18	22.2	0.40 (0.12–1.28)
Single	128	13.3	1.22 (0.63–2.37)	136	27.2	0.69 (0.39–1.22)
Pack Years						
I tertile – low (< 13)	154	13.0	1.00^d^	148	23.6	1.00^d^
II tertile	160	18.8	1.13 (0.54–2.35)	137	28.5	**2.26 (1.12–4.53)**
III tertile – high (≥28.35)	204	8.3	0.45 (0.19–1.05)	131	47.3	**7.28 (3.19–16.6)**
*p* for trend			**0.033**			**< 0.001**
Year of stop smoking				–	–	–
Before 2018	286	4.2	1.00^d^	–	–	–
After 2018	246	21.1	**7.22 (3.53–14.8)**	–	–	–

Distribution of the 539 ex-smokers before lockdown and the 416^a^ current smokers before lockdown according to a worsening of smoking habits (relapse for ex-smokers before lockdown or increase in number of cigarettes per day for current smokers before lockdown) due to the COVID-19 lockdown: overall and by socio-demographic and smoking-related characteristics. Corresponding odds ratios^b^ (ORs) and 95% confidence intervals (CIs). (northern Italy, 2020)

*SCS* Smoking Cessation Service, *GHQ* General Health Questionnaire, *STAI* State-Trait Anxiety Inventory

^a^Of the total 423 current smokers before lockdown, seven subjects did not report their change in smoking habit during lockdown

^b^Estimated by unconditional logistic regression models after adjustment for sex, age, group of participants (SCS; healthcare provider; students); estimates in bold are those statistically significant at the 0.05 level

^c^The sum does not add up to the total because of a few missing values

^d^Reference Category

Among the current smokers before lockdown (Table 3), a self-reported increased number of cigarettes per day during lockdown was associated with being female (compared to male, OR = 1.56; 95% CI, 1.00-2.44) and increasing pack-years (*p* for trend < 0.001). No statistically significant relationship was found between age, marital status and group of participants.

Table 4 shows the distribution of ex-smokers and current smokers before lockdown according to a self-reported worsening of their smoking habit due to the COVID-19 lockdown by psychological indicators. We found that relapse was more frequently reported with decreasing quality of life (*p* for trend < 0.001), decreasing sleep quality (*p* for trend = 0.033) and high levels of anxiety during lockdown (*p* for trend < 0.001). An increase in smoking intensity was more frequently observed with worsening quality of life (*p* for trend < 0.001), worsening sleep quality (*p* for trend < 0.001) and anxiety levels (*p* for trend < 0.001) (Table 4).

**Table 4:** Distribution of the 539 ex-smokers and the 416^a^ current smokers before lockdown (multiple unconditional logistic regression analysis)

Characteristics	Total number of ex-smokers before lockdown^**c**^	Relapsing during lockdown		Total number of current smokers before lockdown^**c**^	Increasing n° of cigarettes per day during lockdown	
		%	OR^b^ (95% CI) relapse vs not relapse		%	OR^b^ (95% CI) Increase vs not increase
Total	539	13.2		416^a^	32.7	1.00^d^
Mood						
Improved	207	14.0	1.00^d^	174	37.9	1.00^d^
Not Modified	220	11.8	0.85 (0.48–1.51)	171	26.3	**0.57 (0.36–0.92)**
Worsened	103	13.6	0.97 (0.48–1.93)	69	33.3	0.85 (0.47–1.55)
GHQ score						
0–10 (high quality of life)	122	5.7	1.00^d^	93	10.8	1.00^d^
11–15	163	10.4	1.94 (0.77–4.87)	129	31.0	**3.59(1.68–7.66)**
16–20	109	15.6	**3.27 (1.29–8.33)**	90	45.6	**6.88 (3.13–15.1)**
> 20 (low quality of life)	92	26.1	**6.17 (2.49–15.3)**	54	50.0	**8.61 (3.62–20.5)**
*p* for trend			**< 0.001**			**< 0.001**
Sleep quality						
I tertile (good)	61	8.2	1.00^d^	50	16.0	1.00^d^
II tertile (average)	287	11.5	1.42 (0.53–3.82)	218	26.6	1.81 (0.80–4.12)
III tertile (poor)	188	17.0	2.33 (0.86–6.32)	145	48.3	**4.73 (2.06–10.9)**
*p* for trend			**0.033**			**< 0.001**
STAI						
I tertile – low (< 43)	180	6.7	1.00^d^	131	20.6	1.00^d^
II tertile	145	10.3	1.60 (0.72–3.56)	123	30.1	1.64 (0.92–2.92)
III tertile – high (≥52)	188	20.7	3.59 (1.80–7.16)	149	46.3	**3.26 (1.89–5.61)**
*p* for trend			**< 0.001**			**< 0.001**

Distribution of the 539 ex-smokers before l0ockdown and the 416^a^ current smokers before lockdown according to a reported worsening of smoking habit (relapse for ex-smokers before lockdown or increase in number of cigarettes per day for current smokers before lockdown) due to the COVID lockdown by psychological indicators. Corresponding odds ratios^b^ (ORs) and 95% confidence intervals (CIs). (northern Italy, 2020)

*SCS* Smoking Cessation Service, *GHQ* General Health Questionnaire, *STAI* State-Trait Anxiety Inventory

^a^Of the total 423 current smokers before lockdown, seven subjects did not report their change in smoking habit during lockdown

^b^Estimated by unconditional logistic regression models after adjustment for sex, age, group of participants (SCS; healthcare provider; students); estimates in bold are those statistically significant at the 0.05 level

^c^The sum does not add up to the total because of a few missing values

^d^Reference category

### Improvement in smoking habit in current smokers before lockdown

Among the 416 current smokers before lockdown, 23.6% self-reported to have improved their smoking habit; in particular, 10.1% stated to have quit smoking and 13.5% to have decreased the number of cigarettes per day during lockdown (Table 5).

**Table 5:** Distribution of the 416^a^ current smokers before lockdown according to an improvement in smoking habit (multinomial logistic regression analysis)

Characteristics	Current smokers before lockdown^**c**^	Quitting smoking during lockdown		Still current smokers, but decreasing n° of cigarettes per day during lockdown	
		%	OR^b^ (95% CI) quit vs not quitting and no decrease	%	OR^b^ (95% CI) decrease vs not quitting and no decrease
Total	416^a^	10.1		13.5	
Sex					
Men	160	8.8	1.00^d^	9.4	1.00^d^
Women	256	10.9	1.68 (0.83–3.40)	16.0	1.75 (0.90–3.40)
Age group (years)					
18–39	124	10.5	1.00^d^	22.6	1.00^d^
40–54	147	13.6	0.70 (0.28–1.73)	12.3	0.63 (0.28–1.41)
55+	145	6.2	**0.25 (0.09–0.71)**	6.9	**0.29 (0.11–0.74)**
*p* for trend			**0.005**		**0.008**
Group of participants					
SCSs	215	13.5	1.00^d^	11.6	1.00^d^
Healthcare provider	149	6.1	**0.28 (0.12–0.66)**	9.4	0.50 (0.24–1.06)
Students	52	7.7	0.31 (0.08–1.18)	32.7	1.53 (0.59–3.96)
Marital status					
Married/cohabitants	196	10.2	1.00^d^	11.2	1.00^d^
Divorced/separated	66	6.1	0.56 (0.18–1.80)	12.1	1.04 (0.42–2.57)
Widowed	18	16.6	1.71 (0.41–7.10)	5.6	0.54 (0.06–4.49)
Single	136	11.0	1.07 (0.47–2.44)	18.4	0.78 (0.35–1.78)
Pack Years					
I tertile – low (< 13)	148	9.5	1.00^d^	24.4	1.00^d^
II tertile	137	13.1	0.83 (0.31–2.17)	12.4	**0.37 (0.16–0.85)**
III tertile – high (≥28.35)	131	7.6	0.45 (0.14–1.44)	2.3	**0.05 (0.01–0.21)**
*p* for trend			0.158		**< 0.001**

Distribution of the 416^a^ current smokers before lockdown according to an improvement in smoking habit (i.e., smoking cessation or reduction in number of cigarettes per day) due to the COVID-19 lockdown, overall and by socio-demographic and smoking-related characteristics. Corresponding odds ratios^b^ (ORs) and 95% confidence intervals (CIs). (northern Italy, 2020)

*SCS* Smoking Cessation Service

^a^Of the total 423 current smokers before lockdown, seven subjects did not report their change in smoking habit during lockdown

^b^Estimated by multinomial logistic regression models after adjustment for sex, age, group of participants (SCSs; healthcare provider; students); estimates in bold are those statistically significant at the 0.05 level

^c^The sums does not add up to the total because of a few missing values

^d^Reference Category

Quitting smoking decreased with increasing age (*p* for trend = 0.005) and was less frequent among the HPs compared to the SCS participants (OR = 0.28; 95% CI, 0.12-0.66).

A decrease in the number of cigarettes smoked per day was less frequently observed with increasing age (*p* for trend = 0.008) and pack-years (*p* for trend < 0.001) (Table 5) and with intermediate anxiety levels (OR = 2.27; 95% CI, 1.04-4.95) (Supplementary Table S1).

### COVID-19 related stressors

Focusing on stressors (Table 6), we analyzed their relationship with a change in smoking habit.

**Table 6:** Distribution of the 539 ex-smokers and the 416^a^ current smokers before lockdown

Characteristics	Worsening in smoking habit (multiple unconditional logistic regression analysis)^**b**^		Improvement in smoking habit (multinomial logistic regression analysis)^**c**^	
	Relapsing 13.2% of 539 subjects	Increasing n° of cigarettes per day 32.7% of 416^**a**^ subjects	Quitting smoking 10.1% on 416^**a**^ subjects	Still current smokers, but Decreasing n° of cigarettes/ per day 13.5% of 416^**a**^ subjects
	OR (95% CI) relapse vs not relapse	OR (95% CI) increase vs not increase	OR (95% CI) quit vs not quitting and no decrease	OR (95% CI) decrease vs not quitting and no decrease
**Stressors related to COVID-19**				
Fear of being infected and getting sick -^d^	**2.42 (1.04–5.64)**	**2.03 (1.11–3.70)**	2.62 (0.73–9.34)	0.78 (0.36–1.66)
Fear of infecting others -^d^	1.74 (0.94–3.20)	**2.00 (1.18–3.38)**	0.96 (0.41–2.25)	0.93 (0.45–1.96)
Sense of powerlessness in protecting oneself from contagion -^d^	**2.28 (1.13–4.58)**	**2.53 (1.46–4.40)**	2.28 (0.85–6.12)	0.82 (0.39–1.72)
Sense of powerlessness in protecting loved ones from contagion -^d^	1.80 (0.98–3.31)	**2.71 (1.60–4.58)**	0.94 (0.41–2.14)	1.03 (0.50–2.15)
Fear of losing loved ones due to virus -^d^	1.78 (0.90–3.51)	**4.18 (2.14–8.15)**	1.12 (0.43–2.92)	1.03 (0.45–2.37)
Fear of dying due to the virus -^d^	**2.24 (1.15–4.34)**	**2.45 (1.44–4.19)**	1.63 (0.66–4.04)	0.96 (0.48–1.95)
Anxiety in listening to news of the epidemic -^d^	**3.62 (1.74–7.50)**	**1.90 (1.13–3.22)**	1.31 (0.57–3.04)	1.27 (0.61–2.64)
**Stressors due to isolation**				
Impossibility/ fear of approaching health facilities while needed -^d^	**1.86 (1.03–3.37)**	1.25 (0.74–2.13)	1.77 (0.76–4.09)	0.81 (0.38–1.74)
Fear of separating from loved ones for the quarantine regime -^d^	1.59 (0.86–2.92)	1.64 (0.96–2.81)	2.39 (0.91–6.27)	1.97 (0.82–4.69)
Sense of powerlessness due to isolation -^d^	**2.79 (1.48–5.27)**	**1.98 (1.18–3.34)**	0.64 (0.27–1.49)	0.99 (0.47–2.09)
Boredom due to isolation^d^	**2.74 (1.47–5.10)**	1.40 (0.82–2.38)	0.83 (0.34–1.99)	1.33 (0.60–2.93)
Feelings of loneliness due to isolation^d^	**3.12 (1.69–5.78)**	**1.73 (1.01–2.96)**	1.04 (0.47–2.30)	1.07 (0.51–2.26)
**Social issues resulting from lockdown**				
Fear of being judged by the neighbors for exits dictated by reasons of need/work/etc. (^e^)	**2.39 (1.40–4.07)**	1.12 (0.71–1.75)	0.73 (0.35–1.50)	0.70 (0.37–1.32)
Fear of being socially excluded due to the origin of regions affected by the virus (^e^)	1.42 (0.84–2.39)	1.30 (0.85–1.99)	0.90 (0.46–1.77)	**0.51 (0.27–0.96)**
Worsening of family relationships during quarantine (^e^)	**2.29 (1.35–3.88)**	**1.86 (1.22–2.84)**	0.82 (0.42–1.62)	0.78 (0.42–1.42)
Fear that nothing will be like before lockdown (9 March, 2020) ^d^	1.80 (0.87–3.76)	**3.16 (1.65–6.04)**	1.94 (0.61–6.21)	0.67 (0.30–1.52)
**Professional and school issues**				
Fear of losing livelihoods/being fired (^e^)	**2.00 (1.17–3.42)**	1.05 (0.66–1.67)	1.17 (0.58–2.35)	0.81 (0.42–1.57)
Reduction IN work and economic opportunities due to the need to stay at home with children because the closure of schools (^e^)	**1.72 (1.01–2.94)**	0.62 (0.37–1.03)	0.82 (0.38–1.77)	0.95 (0.48–1.89)
Reduction in economic opportunities for redundancy fund (for company limitations from COVID-19) (^e^)	1.29 (0.76–2.20)	1.37 (0.87–2.15)	1.04 (0.51–2.10)	1.19 (0.63–2.25)
Difficulty in managing children and teaching online (^e^)	1.04 (0.59–1.83)	0.80 (0.48–1.35)	0.97 (0.45–2.13)	0.94 (0.48–1.85)
Difficulty in adapting to smart working (^e^)	1.14 (0.64–2.04)	1.54 (0.94–2.52)	1.69 (0.82–3.48)	0.99 (0.50–1.97)

Distribution of the 539 ex-smokers before lockdown and the 416^a^ current smokers before lockdown according to a self-reported worsening of smoking habit (relapse for ex-smokers before lockdown or increase in the number of cigarettes per day for current smokers before lockdown) and a self-reported improvement in smoking habit (i.e., smoking cessation or reduction in the number of cigarettes per day) due to the COVID-19 lockdown, overall and by selected stressors. Corresponding odds ratios (ORs) and 95% confidence intervals (CIs). (northern Italy, 2020)

*SCS* Smoking Cessation Service, *GHQ* General Health Questionnaire, *STAI* State-Trait Anxiety Inventory

^a^Of the total 423 current smokers before lockdown, seven subjects did not report their change in smoking habit during lockdown

^b^Estimated by unconditional logistic regression models after adjustment for sex, age, group of participants (SCSs; healthcare provider; students); estimates in bold are those statistically significant at the 0.05 level

^c^Estimated by multinomial logistic regression models after adjustment for sex, age, group of participants (SCSs; healthcare provider; students); estimates in bold are those statistically significant at the 0.05 level

^d^III vs I tertile

^e^Present vs absent

Regarding relapses, in the first category, stressors related to COVID-19, we found four out of seven significant values with an OR range between 2.24 and 3.62; in the second category, stressors due to isolation, we found four out of five significant values with an OR range between 1.86 and 3.12; in the third category, social issues resulting from lockdown, we found two out of four significant values with an OR range between 2.29 and 2.39; in the fourth category, professional and school issues, we found two out of five significant values with an OR range between 1.72 and 2.00.

Regarding the self-reported increase in the number of cigarettes per day, in the first category, stressors related to COVID-19, we found that all stressors were significant with an OR range between 1.90 and 4.18; in the second category, stressors due to isolation, we found two out of five significant values with an OR range between 1.73 and 1.98; in the third category, social issues resulting from lockdown, we found two out of four significant values with an OR range between 1.86 and 3.16; in the fourth category, professional and school issues, no stressors were found to be significant.

### Desire to quit smoking in current smokers during lockdown

The desire to quit smoking was investigated among 441 current smokers during lockdown (Supplementary Table S2). Overall, in 28.6% of the current smokers the desire of quitting was less present, in 36.2% was not modified, and in 35.2% of respondents it was more present during lockdown compared to before lockdown. Students were the subgroup of participants who reported higher percentages of the desire to quit smoking (50.0%) compared to SCSs (34.9%) and HPs (30.8%).

The desire to quit smoking appears to be inversely related to an increase number of cigarettes smoked per day. Subjects smoked less during lockdown, thus they improving their smoking habit, and wanted to quit smoking more (51.8%) compared to those increasing the number of cigarettes per day (44.8%).

## Discussion

Overall, our data suggest that the COVID-19 lockdown resulted in both: a self-reported worsening (13.2% relapsed and 32.7% increased smoking, on average, by six cigarettes per day) and improvement of smoking status (10.1% quit smoking and 13.5% decreased the number of cigarettes per day). These results seem to be in line with existing literature: to date, the research evidence is ambiguous in explaining the effect of COVID-19 lockdown on smokers and looking like it can induce both a worsening and an improvement in smoking habits [20, 30] . To further contribute to this research, we aimed to examine how different factors could be associated to these changes in smoking habits.

To deal with this topic, we used a cross-sectional study design consistent with our research goals, even if it presents different limitations. Due to this design, we could not infer causality from our data because our survey was administered at one time point; thus, we do not have an indication about the causal sequence of events. Moreover, a sample size was not calculated a priori and the enrollment methodology used (convenience sample) did not allow us to obtain a representative sample of ever smokers and to derive conclusions scalable to the general Italian population. For specific subgroups, such as widowed, the estimates were based on few subjects only. These findings should therefore be confirmed by larger studies. Moreover, we did not investigate stressors as mediators between lockdown and changes in smoking habit through a mediation analysis. Considering these limitations, this analysis enabled us to investigate how different and potentially vulnerable subgroups of the Italian population reacted to the COVID-19 lockdown. Our results suggest early hypotheses for future longitudinal research, whose purpose will be to increase the evidence of the associations between exposure to the COVID-19 pandemic and smoking habit.

We considered three different smokers’ groups: SCS’ patients, HP and nursing students. We found no differences between the three groups except for smoking improvement: HPs quit smoking less than the others. This result was confirmed in other studies: Italian doctors are not so likely to quit smoking [31] and healthcare providers exposed to SARS-CoV-2-infected patients presented a higher risk of showing symptoms of anxiety, depression, sleep disorders and psychological distress [32, 33] and an increased difficulty in reducing or quitting smoking [34] in this pandemic period.

Starting from our data, smoking history and some specific consequences of lockdown seem to play an important role.

Regarding smoking history, the time of quitting was found to be the factor that induced more vulnerability in relapse: subjects who quit smoking for a year or less relapsed more than others. This evidence is supported by experimental research, i.e. event-related potential’ *(*ERP) studies: ex-smokers of an average of 1.4 years display the same (low) level of processing bias as never-smokers; thus, they are less vulnerable to a reaction to smoking related stimuli [35]. From clinical research we know that ex-smokers remain at risk of relapse throughout their lives without the extraordinary distress caused by the pandemic: half of new ex-smokers relapses within the first year of cessation [30], but those remain smoke-free for at least 12 months have a 35% chance of relapse throughout the course of their lives [36, 37]. Treatment of chronic tobacco addiction after cessation in ex-smokers has been extensively investigated and remains a challenge: which is one of reasons why in SCS long-term counseling is always recommended for at least 6 months and preferably for 12 months, or even longer during this period. This support could now be provided in different forms to the general population (i.e. online or telephone counseling, automatic messages, educational videos) and should contain specific information about the importance of maintaining smoking cessation status during the COVID-19 pandemic.

Still on smoking history, we found an apparent contradiction: more frequent smokers were more likely to increase the number of cigarettes smoked per day during the lockdown, but, as the pack-years increased, relapses among ex-smokers decreased. It is known that smokers with a smoking history greater than 20 pack-years present significantly higher scores of physical dependence, pleasure of smoking and automatism [38]; therefore they are more inclined to worse their smoking behavior than those who smoke less. However, higher resistance to relapse in former heavy smokers has also been reported, albeit less frequently [39]. One possible explanation is that ex-smokers with a history of heavy addiction internalized the importance (and the difficulty) of quitting and the damage they inflict on themselves when relapsing. In any case, these data should encourage smokers and smoking cessation operators to believe in the utility of promoting smoking cessation even in the most addicted smokers.

Regarding protective factors, in our sample the duration of addiction is likely to play a role: younger ex-smokers (ex-smokers under 40 and students) suffered less from the increased desire to smoke; at the same time among students, the desire to quit was present to a greater extent. These data suggest that young people are more susceptible to attempts to quit and are less prone to relapse; studies from tobacco industries [40], conducted for the opposite target, have underlined that young people have both the highest propensity to quit and receive the most potential benefits from smoking cessation. Furthermore, the lockdown not only negatively influenced smokers and ex-smokers: Jackson found that lockdown was not associated with a significant change in smoking prevalence, but with increases in cessation and quit attempts by smokers [30]. Our data show that smokers under 55 years of age with medium levels of anxiety during lockdown and fear of becoming sick self-reported to have improved their health by completely quitting or reducing cigarettes uses. This result is in line with studies highlighting the functionality of average anxiety and fear, as they help one to prepare for action and to functionally adapt to the living environment [41]. The COVID-19 pandemic, associated with functional distress rates in our populations can therefore represent a circumstance leading individuals to positive behavior change; targeted communication and informative and antismoking programs can produce a useful “teachable moment” [42]. In line with other studies that underline a worsening in smoking habit and wellbeing during lockdown [43–46], our data suggest that the COVID-19 lockdown in this populations has also been a source of high distress, high levels of anxiety and reduced perception of quality of life. The psychological stress, a nonspecific organic response to stressful situations accompanied by physical and psychological symptoms, [47] has an important role in inducing unsanitary living conditions, such as tobacco use [48]. In fact, it is known that a relation between smoking and stress exist [21]. A novel human laboratory model indicated that smokers were less able to gave up cigarettes, smoked more intensely and detected greater gratification and reward from smoking afterward a stress induction, relative to the neutral condition [49]. Hypothalamus–pituitary–adrenal (HPA) axis reactivity, physiologic reactivity, tobacco craving and negative emotion increased significantly due to stress induction [49].

Carreras’ research in Italian smokers [46] showed that the worsening in smoking habit was associated with an increase in mental distress during the COVID-19 lockdown.

In our study, the stressors related to lockdown that mostly involved an increase in cigarette consumption were those related to COVID-19: the fear of being infected and infecting others, and the fear of dying from it. A significant relationship was previously identified between fear and stress from COVID-19, anxiety and depression, as well as significant effects from being sick and having friends or family members who are infected or have died [50]. In addition, among tobacco smokers, there is a perception of an increased vulnerability of their health [51]; this increases the psychological impact of the fear of COVID 19 and, in a vicious circle, favors an increase in smoking, even if smoking exposes the person to an even greater risk from COVID-19.

Among the specific stressors related to COVID-19, the anxiety associated by listening to the news regarding the pandemic has, in our sample, the greatest effect on smoking relapse. The news regarding the fast and wide spread of both accurate and inaccurate information about the COVID-19 pandemic, called an “infodemic”, is considered by the WHO as a problem to manage [52] because of the physical and mental health risks poses for people. Both the continuous exposure to news related to the pandemic and how the news is provided, aimed at increasing the emotional impact, have produced psychological damage, and favored relapses in the context of smoking. It is already known that during the COVID-19 outbreak, news media played an important role in triggering anxiety in people who regularly read relevant news [53, 54]. The WHO [55] guidelines for coping with stress during the COVID-19 outbreak suggest limiting worry and agitation by lessening the time spent watching or listening to media spreading news perceived as upsetting.

In this completely new and upsetting situation social media collaborated to worsen people’s state of mind, but, now more than ever, technology and social media are being used on a massive scale to keep people safe, informed, productive and connected [52]. Social network and video call systems constituted a real lifeline for people isolated because of illness or physical distancing requirement. In all social networks, pages and group dedicated to self-help support of ex-smokers were created, and professional anti-smoking, and relapse prevention interventions have been tested. The effectiveness of these tools in preventing relapses is not yet fully known [56], but the helpfulness of social networks’ support has been demonstrated by the number of page visits and interactions [57].

The relationship between smoking and psychiatric symptoms is a studied issue [58, 59]. Anxiety is one of the psychiatric disorders most related to smoking and increased susceptibility to stress: many smokers wrongly believe that smoking helps to relieve stress, so they consider smoking as an instrument to manage high stress’ levels [60]. However, a systematic review concluded that it was not possible to identify a unique model to consider the relationship between anxiety and smoking and that a bidirectional relationship between the two is possible [61]. Smoking and anxiety have a bidirectional relationship as they influence each other. So, it is important to spread that smoking is also a cause of increased anxiety and that quitting can reduce it with a consequent wellness improvement. Simultaneously, promoting alternative stress and anxiety management strategies may be useful in preventing relapses and an increase in the number of cigarettes.

Further studies are needed to better define the role of the various factors present in this complex situation, but it is possible to state that it would be useful for governments to pay attention to the physical and psychological health consequences deriving from the restrictions related to the COVID-19 pandemic by implementing information campaigns on stress management and telephone and online support channels because this has an important impact on the quality of life, lifestyle and health.

## Conclusion

The lockdown during the COVID-19 pandemic created a highly stressful situation for ever-smokers and relapse or increasing in the number of cigarettes smoked have been associated with it. Despite this, smoking cessation still occurred in this period, particularly in the younger participants of our sample.

## Supplementary Information

**Additional file 1: Table S1.** Distribution of the 416* current smokers before lockdown (multinomial logistic regression analysis). **Table S2.** Distribution of the 441 current smokers* during lockdown according to their desire to quit smoking.

## Data Availability

The datasets generated and/or analysed during the current study are not publicly available because we prefer that these data are not made public to avoid any type of exploitation by external companies but are available from the corresponding author on reasonable request.
