# Supplementary material for: Factors associated with a change in smoking habit during the first COVID-19 lockdown: an Italian cross-sectional study among ever-smokers
Source: BMC Public Health. 2022 May 25;22:1046. doi: 10.1186/s12889-022-13404-5 (PMC9132352; doi:10.1186/s12889-022-13404-5)
Supplement: Supplementary file 1 — Additional file 1: Table S1. Distribution of the 416* current smokers before lockdown (multinomial logistic regression analysis). Table S2. Distribution of the 441 current smokers* during lockdown according to their desire to quit smoking. [file 12889_2022_13404_MOESM1_ESM.docx]

**Supplementary Materials**

**Table S1. Distribution of the 416* current smokers before lockdown (multinomial logistic regression analysis)**

| **Characteristics** | **Current smokers before lockdown***** | **Quitting smoking during lockdown** | | **Still current smokers, but decreasing n° of cigarettes per day during lockdown** | |
| --- | --- | --- | --- | --- | --- |
|  |  | % | **OR (95% CI)**  **Quit vs not quit and no decrease** | **%** | **OR (95% CI)**  **Decrease vs not quit and no decrease** |
| Total | 416* | 10.1 |  | 13.5 |  |
| Mood |  |  |  |  |  |
| Improved | 174 | 9.8 | 1.00^ | 12.1 | 1.00^ |
| Not Modified | 171 | 9.4 | 1.20 (0.56-2.55) | 14.0 | 1.72 (0.87-3.41) |
| Worsened | 69 | 13.0 | 1.23 (0.50-3.04) | 15.9 | 1.55 (0.67-3.58) |
| GHQ score ° |  |  |  |  |  |
| 0-10 (high quality of life) | 99 | 12.2 | 1.00^ | 13.1 | 1.00^ |
| 11-15 | 146 | 11.0 | 0.95 (0.41-2.20) | 14.4 | 1.12 (0.51-2.49) |
| 16-20 | 107 | 8.4 | 0.55 (0.21-1.46) | 11.2 | 0.69 (0.28-1.69) |
| >20 (low quality of life) | 64 | 7.8 | 0.46 (0.15-1.46) | 15.6 | 0.99 (0.38-2.58) |
| p for trend |  |  | 0.101 |  | 0.641 |
| Sleep quality |  |  |  |  |  |
| I tertile (good) | 50 | 12.0 | 1.00^ | 10.0 | 1.00^ |
| II tertile (average) | 218 | 11.5 | 1.07 (0.40-2.90) | 15.1 | 2.12 (0.73-6.13) |
| III tertile (bad) | 145 | 7.6 | 0.58 (0.19-1.72) | 12.4 | 1.38 (0.45-4.18) |
| p for trend |  |  | 0.183 |  | 0.902 |
| STAI ° |  |  |  |  |  |
| I tertile – low (<43) | 133 | 13.6 | 1.00^ | 9.0 | 1.00^ |
| II tertile | 128 | 7.8 | 0.52 (0.22-1.23) | 20.3 | **2.27 (1.04-4.95)** |
| III tertile – high (≥52) | 155 | 9.0 | 0.50 (0.23-1.10) | 11.6 | 1.10 (0.49-2.49) |
| p for trend |  |  | 0.087 |  | 0.972 |

Distribution of the 416 current smokers before lockdown according to an improvement in smoking habit (i.e., smoking cessation or reduction in number of cigarettes per day) due to the COVID-19 lockdown, overall and by psychological indicators. Corresponding odds ratios** (ORs) and 95% confidence intervals (CIs). (northern Italy, 2020).

GHQ: General Health Questionnaire; STAI: State-Trait Anxiety Inventory

* Of the total 423 current smokers before lockdown, seven subjects did not report their change in smoking habit during lockdown.

** Estimated by multinomial logistic regression models after adjustment for sex, age, group of participants (SCS; healthcare provider; students); estimates in bold are those statistically significant at the 0.05 level.

*** The sum does not add up to the total because of a few missing values.

^ Reference Category

Estimates in bold are those statistically significant at the 0.05 level

**Table S2. Distribution of the 441 current smokers* during lockdown according to their desire to quit smoking**

| **Characteristic** | **N** | **Desire to quit smoking** | | |  |
| --- | --- | --- | --- | --- | --- |
|  |  | **Less present**  **%** | **Not Modified**  **%** | **More present**  **%** | ***p*-Value** |
| Total | 441* | 28.6 | 36.2 | 35.2 |  |
| Sex |  |  |  |  |  |
| Men | 172 | 27.3 | 38.4 | 34.3 | 0.760 |
| Women | 269 | 29.4 | 34.9 | 35.7 |  |
| Age group |  |  |  |  |  |
| <40 | 124 | 25.8 | 32.3 | 41.9 | 0.372 |
| 40-54 | 157 | 31.9 | 36.3 | 31.8 |  |
| 55+ | 160 | 27.5 | 39.4 | 33.1 |  |
| Group of participants |  |  |  |  |  |
| SCS | 235 | 32.8 | 32.3 | 34.9 | **0.013** |
| Healthcare provider | 156 | 27.5 | 41.7 | 30.8 |  |
| Students | 50 | 12.0 | 38.0 | 50.0 |  |
| Smoking at baseline |  |  |  |  |  |
| No | 67 | 50.8 | 17.9 | 31.3 | **<0.001** |
| Yes | 374 | 24.6 | 39.6 | 35.8 |  |
| Improving (Reducing) |  |  |  |  |  |
| Yes | 56 | 12.5 | 35.7 | 51.8 | **0.004** |
| No | 385 | 30.9 | 36.4 | 32.7 |  |
| Worsening (Increasing or relapsing) |  |  |  |  |  |
| Yes | 203 | 44.8 | 20.2 | 35.0 | **<0.001** |
| No | 238 | 14.7 | 50 | 35.3 |  |

Distribution of the 441 current smokers* during lockdown according to their desire to quit smoking (less present, not modified or more present) due to the COVID-19 lockdown, overall and by selected individual-level characteristics. (northern Italy, 2020).

***** Of the total 452 current smokers during lockdown, 11 provided no information about their desire to quit smoking.
